# Modifiers of Adeno-Associated Virus-Mediated Gene Expression in Implication for Serotype-Universal Neutralizing Antibody Assay

**DOI:** 10.1089/hum.2020.074

**Published:** 2020-10-16

**Authors:** Karina Krotova, George Aslanidi

**Affiliations:** Hormel Institute, University of Minnesota, Austin, Minnesota, USA.

**Keywords:** adeno-associated virus, neutralizing antibodies, selective inhibitor of AMPK, assay development

## Abstract

Adeno-associated virus (AAV)-based gene therapy is undergoing major expansion into clinical practice, with two treatments currently being granted Food and Drug Administration (FDA) approval. However, the presence of pre-existing neutralizing antibodies (NAB) is one of the significant hurdles for the clinical application of AAV vectors that significantly limits the patient population, which benefits from the treatment. A reliable diagnostic to evaluate the patient's seropositivity is required to ensure the effectiveness of the AAV-mediated therapeutic. Here, we describe a simple method for the determination of AAV NAB activity based on our finding that Compound C makes HEK293 cell highly permissive for infection by 10 commonly used AAV serotypes.

## Introduction

Adeno-associated virus (AAV) has been widely recognized as a safe and effective clinical-stage vector for gene therapy in a broad spectrum of inherited diseases such as Leber's congenital amaurosis, hemophilia A and B, and muscular dystrophy.^1–3^ Most recently, AAV-based treatments had a major ground break as two gene therapies for rare eye disease and spinal muscular atrophy were approved by the US Food and Drug Administration (FDA) and became available for patients.^4,5^

However, AAV seropositivity of the general patient population, due to natural virus infection and the presence of neutralizing antibodies (NAB) to commonly used AAV serotypes, limits the cohort of successful recipients of novel therapies.^6–13^ For that reason, numerous attempts were made to identify or develop novel AAV serotypes that can avoid neutralization.^14–21^ Nevertheless, regardless of the successful use of novel AAV capsid variants in animal models, they required additional preclinical evaluation before use for human application.

Thus, a reliable method for patients screening to evaluate the presence and activity of NAB for commonly used AAV serotypes is necessary. Several methods based on different principles to determine NAB titer are published, including enzyme-linked immunosorbent assay (ELISA),^22^ a quantitative polymerase chain reaction-based method evaluating AAV binding to cells,^23^ and *in vivo* inhibition of AAV activity by injection of a tested sample in C57BL/6 mice.^24^ Nevertheless, some of the methods, such as measurements of NAB activity using mice, are lengthy and hard to standardize.

An assay based on *in vitro* infection of cells with the AAV encoded reporter gene is easy to establish and reproduce,^25–27^ and it is recommended by the FDA as the assay of choice.^28^ In this assay, cells are transfected with reporter AAV in the presence of serial dilution of the tested sample. The titer of the NAB is determined as a sample dilution at which the transduction efficiency of the reporter gene is half of the maximum value.

Nevertheless, the challenge of identifying an appropriate cell line for all commonly used AAV serotypes^26,29^ and the reporter gene with sufficient sensitivity to detect reliable differences between evaluated serum samples^30^ remains unresolved. For example, AAV8 is a highly efficient serotype for transgene delivery to the liver *in vivo*; at the same time, it is hardly infectious and requires a very high multiplicity of infections (MOI) to transduce cells *in vitro*.

To overcome this limitation, a number of cell lines were used (HEK293, HeLa, Huh7, C2C12, *etc.*) for different AAV serotypes, a wide range (10^3^–10^5^ viral genome (vg)/cell) of AAV MOI, and a large selection of pharmacological drugs previously identified to significantly enhance the transduction of some AAV serotypes *in vitro.*^24,26,31^ In addition, a helper virus, such as Adenovirus, was previously applied to increase expression in AAV-transduced cells.^24^ Also, a recently published detailed protocol of NAB assay suggested using the HEK293-derived cell line 2V6.11 (not commercially available) for AAV8, which expresses the adenovirus E4 ORF gene under the control of the ecdysone-inducible promoter and ponasterone A to make these cells permissive for AAV infection.^27^

This study was designed to develop a protocol for reliable estimation of NAB titer against AAV8, as well as other AAV serotypes, which allow the use of a commonly available cell line such as HEK293. For that purpose, we performed limited screening by infecting HEK293 cells with AAV8-luciferase (Luc) in the presence of several pharmacologically active drugs. We identified a selective inhibitor of AMP-activated protein kinase (AMPK) Dorsomorphin, also known as Compound C (CC), as an enhancer of the infection of HEK293 cells by AAV8, and other AAV serotypes, without cytotoxic effects.

As a result of this significant observation, we developed a protocol to determine NAB titers that works efficiently for all AAV serotypes we tested.

## Protocol Development

Briefly, AAV vectors used in this study (serotypes 1, 2, 3, 5, 6, 7, 8, 9, 10, and recently identified Anc80L65^32^) were packaged in HEK293 cells by triple transfection with polyethyleneimine (PEI) and isolated by an iodixanol four-step gradient followed by ion-exchange column purification as described.^33,34^ This method provides high-purity vector preps with <10% empty capsids.^35,36^ The vectors contained a single-strain expression cassette with a chicken-β-actin promoter (CB)-driven fusion of firefly luciferase (Luc), and yellow fluorescent protein (YFP) genes.^37,38^

**Figure 1.**
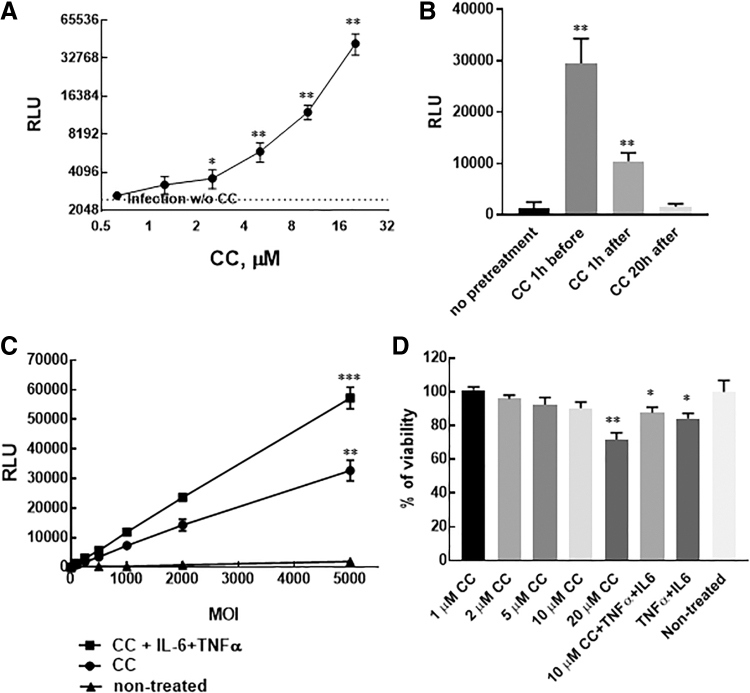
Pretreatment of HEK293 cells with CC significantly increases the infectivity of AAV8-Luc. **(A)** HEK293 cells were treated with different concentrations of CC and 1 h later infected with AAV8-Luc at MOI = 2,000 vg/cell. *p < 0.05 and **p < 0.01 compared with luciferase activity in untreated cells. **(B)** 10 μM CC was added to HEK293 at different time points during infection with AAV8-Luc. **p < 0.01 compared with luciferase activity in untreated cells. **(C)** HEK293 cells were infected with different MOI (100–5,000 vg/cell) of AAV8-Luc in the presence of 10 μM CC or 10 μM CC +20 ng/mL IL-6 + 20 ng/mL TNF-α. Luciferase activity was measured 24 h later. **p < 0.01 for all MOI in the presence of CC compared with infections without pretreatment of HEK293 cells, ***p < 0.01 for MOI in the range 500–5,000 vg/cell in the presence of CC+IL-6+TNF-α compared with the infections in the presence of CC. For MOI = 250 vg/cell, *p < 0.05. **(D)** HEK293 cells were incubated with different concentrations of CC or with 20 ng/mL IL-6 + 20 ng/mL TNF-α for 48 h. At the end of incubation, cell counting reagent CCK-8 (APExBio, Boston, MA) was added to wells for an additional 1 h. The absorbance was measured at 450 nm. The number of cells in treated wells was compared with the number of cells in non-treated control wells, which was considered as 100% viability. *p < 0.05 and **p < 0.01 compared with non-treated controls. AAV, adeno-associated virus; CC, compound C; CCK-8, cell counting kit-8; IL-6, interleukin-6; MOI, multiplicity of infections; TNF-α, tumor necrosis factor alpha.

First, escalation doses of CC were used to identify the optimal drug concentration for AAV serotype 8 (Fig. 1A). Pretreatment of HEK293 cells with CC dose dependently increased luciferase activity 24 h after infection. The dose 10 μM CC was chosen for the next experiments as the minimal concentration that induces high AAV transduction; luciferase activity was five times higher compared with non-treated cells, and values were two orders higher than the background.

In the next step of protocol optimization, 10 μM CC was added to cells at different time points before or after AAV8 infection. The maximum increase in AAV8-mediated luciferase expression was observed if cells were pretreated 1 h before infection (Fig. 1B). Hence, in all subsequent experiments, HEK293 cells were pretreated with 10 μM CC 1 h before infection. Next, we used escalating doses of AAV8-Luc in the presence of CC and demonstrated the linear dose dependence of luciferase expression at a range of MOI from 100 to 5,000 vg/cell.

In addition, we also showed that pretreatment of cells with CC together with pro-inflammatory cytokines interleukin (IL)-6 (20 ng/mL) and tumor necrosis factor alpha (TNF-α) (20 ng/mL) further increased luciferase expression. It should be noted that without CC treatment with IL-6 and TNF-α only marginally increases luciferase expression (data not shown). The treatment of HEK293 cells with CC for 48 h does not induce cytotoxicity at doses up to 10 μM, at 20 μM CC and a combination of 10 μM CC with TNF-α and IL-6 mild cytotoxic effect was observed (Fig. 1D). The dramatic improvement of AAV8 transduction *in vitro* in the presence of CC prompted to test whether a similar effect can be achieved for other AAV serotypes.

**Figure 2.**
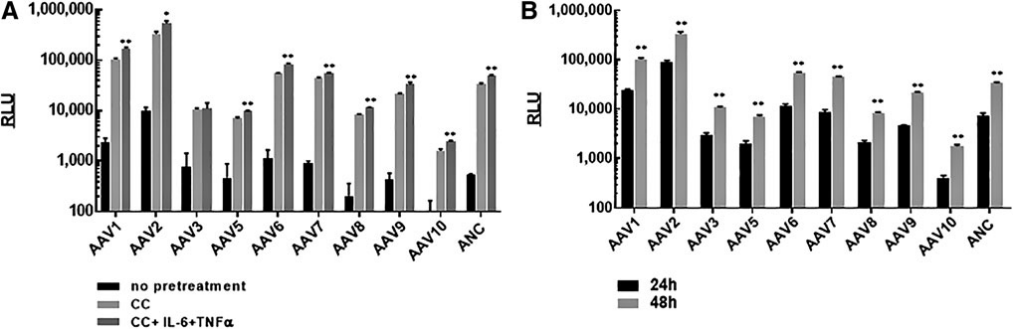
CC enhanced the infectivity of all tested AAV serotypes. **(A)** HEK293 cells were infected with different AAV-Luc serotypes at MOI 2,000 vg/cell in the presence of 10 μM CC, or CC+IL-6+TNF-α. Luciferase activity was measured 48 h later. For all serotypes, luciferase activity was higher in the presence of CC compared with non-treated cells (*p* < 0.01). *p < 0.05 and **p < 0.01 for cells infected in the presence of CC+IL6+TNF-α compared with CC only. **(B)** Comparison of luciferase activity at 24 and 48 h after infection. Cells were infected in the presence of CC as described in **(A)**. *p < 0.05 and **p < 0.01 for 48 h compared with 24 h.

We analyzed the transduction efficiency of 10 common AAV serotypes at MOI 2,000 vg/cell. Without pretreatment, only cells infected with AAV1 and AAV2 had luciferase activity that was significantly higher than the background at 24 h (data not shown) and 48 h after infection (Fig. 2A). At the same time, pretreatment with CC allowed the measurement of sufficient luciferase activity for all tested serotypes (Fig. 2A). In addition, an extension of incubation time after infection from 24 to 48 h significantly increased luciferase activity for all serotypes (Fig. 2B).

Such an extension of the time up to 48 h is beneficial for NAB measurements for hard-to-infect serotypes such as AAV3, 5, 10, and 8. In fact, without CC the luciferase activity for AAV10 was at a background level, and for AAV3, 5, 8, and 9 it was in only a marginally higher background. Thus, it makes it impossible to measure NAB titers for these serotypes in non-treated HEK293 cell. After pretreatment with CC, all these serotypes demonstrated a high level of luciferase expression, which allows to establish NAB titer protocol. The highest luciferase activity was observed for AAV2 (more than 1,000 times higher background in the presence of CC at 48 h) and the lowest was observed for AAV10 (8 times higher background).

We also showed that the addition of IL-6 and TNF-α to CC treatment improved infection efficiency for all serotypes, but not for AAV3 (Fig. 2A). That additional treatment can be used for some serotypes such as AAV10 in case the treatment with CC and increase of MOI still do not provide reporter gene activity enough to set up the NAB assay.

**Figure 3.**
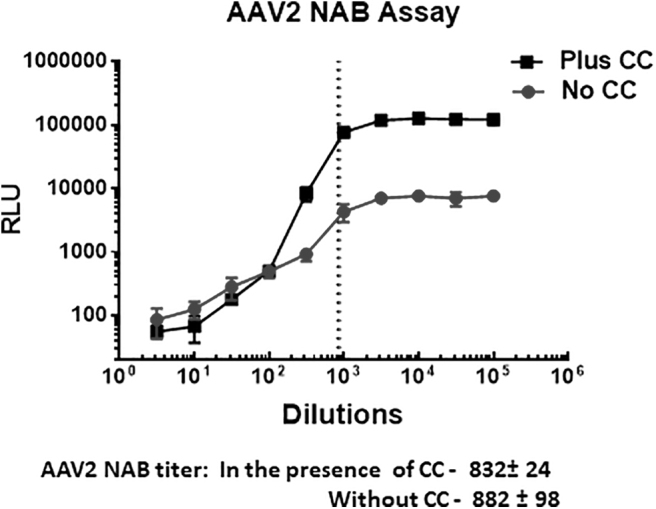
The performance of NAB assay in the presence of CC does not change NAB titer. The mouse plasma was collected 1 month after injection of AAV2 and analyzed in the presence of NAB by utilizing HEK293 cells either pretreated or non-treated with CC. Although CC significantly increased the values for luciferase activity, it did not affect NAB titer (as demonstrated overlaid vertical *dotted lines* corresponded to NAB titers for AAV2 measured in the presence and absence of CC). The observed difference is not statistically significant. NAB, neutralizing antibodies.

Next, we ensure that the addition of CC to media does not affect readout in NAB titer. Thus, we compared the values for AAV2 NAB titer determined in experiments with non-treated and CC pretreated HEK293 cell. AAV2 was chosen as the most infectious serotype for HEK293 cells, and therefore, NAB titers can be measured without additional stimulation of cells. Mouse serum from animals injected intramuscularly (i.m) with 10^10^ vg/mouse of AAV2-Luc was analyzed by the protocol described next. The results shown in Fig. 3 strongly suggest that CC does not change NAB titer and inhibition appears at the same dilution of the serum.

**Figure 4.**
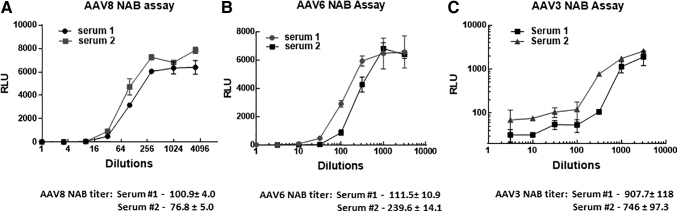
Examples of NAB assay for different serotypes performed with HEK293 cell pretreated with CC. For each AAV, serotype mice were injected with 10^10^ vg/animal and serum was collected 3 weeks later for NAB assay. **(A)** Assay for AAV8. **(B)** Assay for AAV6. **(C)** Assay for AAV3.

Finally, we demonstrate the successful measurements of NAB titers by using the protocol described next for three different AAV serotypes 8, 6, and 3 with mouse serum collected from animals injected i.m. with corresponding AAVs (Fig. 4).

The interaction of AAV vectors with host cells occurs through multiple steps: virus attachment to target cells by binding to receptor and co-receptors, entry by endocytosis, intracellular trafficking, nuclear translocation, capsid uncoating, and vector-mediated gene expression.^39–45^ Investigation into which steps in this complex process CC affect HEK293 cell infection with AAV is out of the scope of this study. Since CC, which at first was identified as an AMPK inhibitor, also inhibits other kinases,^46^ we can speculate that its effect can be partially explained by preventing AAV capsid phosphorylation with these kinases and subsequent AAV degradation by proteasome machinery.^47,48^

In summary, our findings resulted in the development of easily setting up a universal protocol for the analysis of AAV-specific NAB for commonly used serotypes. However, such an enhancement in the transduction efficiency of AAV vectors by CC can be used for the development of other infection-based *in vitro* assays.

## Protocol

The following protocol provides a detailed example of utilizing HEK293 cells pretreated with CC alone or together with TNF-α and IL-6 to estimate the titer of AAV-specific NAB in tested serum samples. In general, AAV vectors expressing luciferase are incubated with serial dilutions of tested serum sample, and they are then added to HEK293 cells pretreated with CC. The expression of luciferase is analyzed 24 or 48 h later in cells by measuring enzyme activity using Bright-Glo luciferase substrate. NAB titer corresponds to the dilution of the test serum sample at which 50% of the luciferase signal is inhibited compared with the “virus only” control.

Of note, the protocol does not include the description of AAV preparation, titration, and quality control. However, these are important steps for the reproducibility of data across the labs and should be taken into consideration before setting up the NAB assay.

## Materials

The essential materials required to complete protocol are listed in Table 1.

**Table 1.** Essential materials required to complete protocol

Reagents	Supplier	Specific Handling	Storage Conditions
Compound C in solution	EMD Biosciences, 171261	Hazardous in case of skin contact (irritant), of eye contact (irritant), use standard procedures to avoid contact with skin and eyes	−20°C
293T cell line	ATCC	Use low passages	In liquid nitrogen
FBS	Thermofisher (Gibco), 16000044	Heat inactivate at 56°C for 30 min before use	−80°C
Dulbecco's modified Eagle's medium	Thermofisher (Gibco), 11965084		+4°C
Reporter AAV/luciferase stocks	Produced according to lab protocol	Store in aliquots to reduce thaw/freeze cycles.	−20°C
Bright Glo luciferase assay system	Promega, E2610, E2620, E2650	The lyophilized Bright-Glo™ Substrate contains DTT and is, therefore, classified as hazardous. The reconstituted reagent is not known to present any hazards, as the concentration of DTT is <1%.	−20°C
Trypsin-EDTA (0.05%), phenol red	Thermofisher (Gibco), 25300120		−20°C or up to 1 week at +4°C
PBS	Thermofisher (Gibco), 20012027		Room temperature

AAV, adeno-associated virus; DTT, dithiothreitol; EDTA, ethylenediaminetetraacetic acid; FBS, fetal bovine serum; PBS, phosphate-buffered saline.

### Supplies

Plates, 96-well with black or white walls and flat, clear-bottom, tissue culture-treated.

Polypropylene or other low-absorption plates, 96-well, U- or V-bottomed, sterile.

### Equipment

CO_2_ incubator.

Automotive cell counter (Countess II; ThermoFisher).

BioTek Synergy Neo 2 or any other spectrophotometer with the capability to read luminescence in multi-well plates.

Multichannel pipette.

### Reagents' preparation

Complete media: Dulbecco's modified Eagle's medium (DMEM) supplemented with 10% fetal bovine serum (FBS) and antibiotics. To 1 L of DMEM add 100 mL of heat-inactivated FBS and 10 mL of 10 × penicillin/streptomycin. Store at 4°C for 1 month.

Test samples: Serum, plasma, or any other biological liquid. Heat inactivate at 56°C for 30 min. Samples can be stored at −80°C in aliquots.

Diluent: FBS should be heat inactivated (HI) at 56°C for 30 min before use as a diluent. Aliquots of HI-FBS can be stored in aliquots at −80°C.

CC is purchased as 10 mM stock solution in DMSO. It is stored in aliquots at −20°C.

Bright Glo Luciferase assay system: Before first use, transfer the contents of one bottle of Bright-Glo Buffer to one bottle of Bright-Glo Substrate. Mix by inversion until the substrate is thoroughly dissolved. Store in aliquots at −20°C. According to the manufacturer's instructions, each aliquot can be subjected to freeze/thaw up to seven times without loss of activity if it is thawed at temperatures below 25°C.

## Experimental Procedure

### Day 0

(1) Remove serum-containing medium from HEK293 cell culture; then, gently wash cells twice with 100 μL of phosphate-buffered saline (PBS). Cells should be at a low passage and ∼80% confluent without being overgrown. Harvest cells by trypsinization and perform a cell count.
(2) Resuspend cells in complete DMEM culture medium at 200,000 cells/mL. Seed cells in 96-well plates with black or white walls and a clear bottom: 20,000 cells/well in 100 μL of complete media. Incubate cells overnight in a CO_2_ incubator at 37°C.

### Day 1

(1) Observe cells in the plate under a microscope. Cells should be 50–80% confluent.

Warm up serum-free DMEM. Thaw the stock solutions of CC (10 mM in DMSO), and analyze reporter AAV-Luc for serotype.

(2) Do not change media in the plate with cells. Activate cells by adding 50 μL/well of serum-free DMEM containing 30 μM CC (note that the final concentration is 10 μM). For example, for the whole 96-well plate, 6 mL of DMEM supplemented with 18 μL CC will be needed.

Put the plate back in a CO2 incubator for 1 h.

(3) Prepare serial dilutions of test samples by using HI-FBS as a diluent in 96-well plates with a U or V bottom.

An example of the dilution strategy is given in Table 2.

**Table 2.** Preparation of the dilution cascade for the test samples (enough for three repeats × 10 μL)

Dilution Factor		Volume of Test Sample	Volume of Diluent (μL)
Dilution 1	1:1	40 μL of undiluted material	0
Dilution 2	1:4	10 μL of dilution 1	30
Dilution 3	1:16	10 μL of dilution 2	30
Dilution 4	1:64	10 μL of dilution 3	30
Dilution 5	1:256	10 μL of dilution 4	30
Dilution 6	1:1,024	10 μL of dilution 5	30
Dilution 7	1:4,096	10 μL of dilution 6	30

Positive control: HI-FBS alone.

HI-FBS, heat-inactivated FBS.

(4) Prepare the working solution of AAV. First, calculate the working concentration of AAV, which is based on the number of cells in wells and multiplicity of infection (MOI) will be used.

Example: If at the day of infection cells are 50% confluent (assuming that 100% confluence consists of 50,000 cells/well) and in each well AAV-Luc will be added at MOI = 2,000 vg/well in 5 μL, the working virus concentration is calculated as follows:


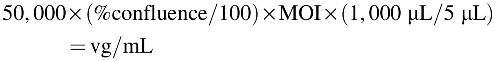


50,000 × (50/100) × 2,000 × 200 = 1 × 10^10^ vg/mL-working concentration of AAV-Luc

For the whole 96-well plate, 800 μL of AAV-Luc plus extra is needed (if to mix 25 μL of AAV for each sample in triplicate, then 96 wells/3 × 25 μL = 800 μL).

If the stock of AAV8-Luc is 1 × 10^12^ vg/mL, it needs to be diluted with PBS to working concentration 1 × 10^10^ vg/mL (dilution factor 100); then for 1 mL of working concentration of AAV, 10 μL of stock AAV-Luc should be diluted with 990 μL PBS.

(5) Mix 25 μL of each dilution of the sample with 25 μL AAV-Luc (ratio 1:1) in new 96-well plates with a U or V bottom and incubate for 1 h at 37°C. For positive control, mix 25 μL AAV-Luc AAV with 25 μL diluent. For negative controls (background), mix 25 μL diluent (HI-FBS) with 25 μL PBS.
(6) Add AAV/sample mix to the plate with CC pretreated HEK293 cells at 10 μL of mix per well. Each sample dilution should be analyzed at least in triplicate. Also include positive control with maximum (MAX) infection level (AAV mixed with diluent HI-FBS), and background wells (no AAV): HI-FBS mixed with PBS (MIN). An example of a plate layout is shown in Table 3.
(7) Wrap the plate in aluminum foil and incubate in a CO_2_ incubator for 24 h.

**Table 3.** Example of assay plate layout

	Sample 1			Sample 2			Sample 3			Sample 4		
	1	2	3	4	5	6	7	8	9	10	11	12
A	1:1	1:1	1:1	1:1	1:1	1:1	1:1	1:1	1:1	1:1	1:1	1:1
B	1:4	1:4	1:4	1:4	1:4	1:4	1:4	1:4	1:4	1:4	1:4	1:4
C	1:16	1:16	1:16	1:16	1:16	1:16	1:16	1:16	1:16	1:16	1:16	1:16
D	1:64	1:64	1:64	1:64	1:64	1:64	1:64	1:64	1:64	1:64	1:64	1:64
E	1:256	1:256	1:256	1:256	1:256	1:256	1:256	1:256	1:256	1:256	1:256	1:256
F	1:1,024	1:1,024	1:1,024	1:1,024	1:1,024	1:1,024	1:1,024	1:1,024	1:1,024	1:1,024	1:1,024	1:1,024
G	1:4,096	1:4,096	1:4,096	1:4,096	1:4,096	1:4,096	1:4,096	1:4,096	1:4,096	1:4,096	1:4,096	1:4,096
H	MAX	MAX	MAX	MAX	MAX	MAX	MIN	MIN	MIN	MIN	MIN	MIN

MAX, maximum AAV infection level: mixed with diluent (HI-FBS); MIN, background levels (no AAV): HI-FBS mixed with PBS.

### Day 2

(1) Thaw an aliquot of Bright Glo Luciferase assay reagent at room temperature in the dark. For one 96-well plate 6 mL of reagent is sufficient.
(2) Remove the plate from a CO_2_ incubator. Dump media from the plate by turning it upside down quickly. Tap the rest of the media on paper towels. Fill the plate with 50 μL PBS/well.
(3) Set up a spectrophotometer to read chemiluminescence. If the spectrophotometer is BioTek, the optimal read conditions are: 500 ms, gain 125–135. Add 50 μL of Bright Glo substrate to each well, incubate the plate for 3 min in the dark, and finally read. For maximal light intensity, samples should be measured within 15 min of reagent addition.

### Calculation of anti-AAV NAB titer

#### First method

NAB titer is defined as the neutralizing titer of the sample and is the first dilution at which 50% or greater inhibition of the luciferase expression is measured.

It can be quantified manually by subtracting average background values from all measurements and then by calculating the percent of the total luciferase expression:


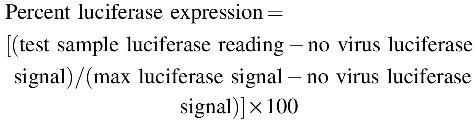


The first dilution of the sample with 50% or greater inhibition of the luciferase activity is used to determine the neutralizing titer. For example, if 50% or greater inhibition is observed at a 1:10 dilution of the sample, the titer is reported as 10.

#### Alternative method

Another way to determine NAB titer is by using GraphPad Prizm or any other suitable software. Place data as an XY file, where X is testing sample dilutions and Y is testing luminescence measurements in triplicate. Then, go to the results folder to analyze data: Use XY analysis→Nonlinear regression (curve fit)→[Agonist] versus response→Variable slope (four parameters). It will calculate EC50, which corresponds to NAB titer. Note that the use of this analysis depends on the appropriate inhibition curve with well-defined plateaus at the minimum and maximum dilutions. In the case when the values at the highest dilution of a tested sample are much lower than the maximum value, the assay should be repeated with more diluted samples.

## Troubleshooting

A summary of critical steps of the protocol with possible troubleshooting is explained in Table 4.

**Table 4.** Summary of critical steps of the protocol with possible troubleshooting

Problem	Solution
High variability in readout across triplicate wells	The major source of such variability is unequal number of cells in the different wells. The HEK293 cell is readily detached from the plate during the trypsinization step, but it does not dissociate easily from each other. Ensure that cells form a single-cell suspension at the step of plating. In addition, wrapping the plate in aluminum foil during incubation time in a CO_2_ incubator will help to maintain even temperature across the plate and as result more even growing.
	Since HEK293 cells detach easily, to prevent the loss of cells during the assay, avoid aspiration of media or plate washing.
Low level of luciferase readout	The aliquot of reporter AAV lost activity or the titer was miscalculated. Take another aliquot or re-titer virus.
	The quality of the HEK293 cell is also very important. Cells should be of low passage and be 50–70% confluent at the beginning of the experiment.
	To increase the signal for serotypes with low infectivity, several approaches can be utilized. (1) Time of incubation can be extended from 24 to 48 h. (2) Multiplicity of infections can be increased. However, make sure that luciferase signal is dose dependent and is not saturated. (3) For many serotypes, pretreatment of HEK293 cell with Compound C together with interleukin-6 and tumor necrosis factor alpha will additionally increase the luciferase readout.
The RLU of MAX luciferase signal is significantly lower than some of the dilutions of the test sample	FBS is used as a diluent and it inhibits AAV infection by itself. Different providers and a lot of FBS should be tested on the ability to affect the AAV infectivity.
